# Spontaneous splenic rupture in an active duty Marine upon return from Iraq: a case report

**DOI:** 10.1186/1752-1947-4-353

**Published:** 2010-11-05

**Authors:** Jason P Rice, Christian M Sutter

**Affiliations:** 1 Branch Medical Clinic, Marine Corps Air Station Miramar, 19871 Mitscher Way, San Diego, CA 92145, USA; 2 Naval Health Clinic Great Lakes, Department of Family Medicine, 3001A Sixth Street, Great Lakes, IL 60088, USA

## Abstract

**Introduction:**

Atraumatic splenic rupture is a rare event that has been associated with several infectious disease processes. In the active duty military population, potential exposure to these pathogens is significant. Here we discuss the case of an active duty Marine with spontaneous splenic rupture upon return from a six-month deployment in Iraq.

**Case presentation:**

A previously healthy 30-year-old Caucasian male active duty Marine presented with abdominal pain, fever and diarrhea after deployment to Iraq in support of Operation Iraqi Freedom. Based on clinical and radiographic evidence, a diagnosis of spontaneous splenic rupture was ultimately suspected. After exploratory laparotomy with confirmation of rupture, splenectomy was performed, and the patient made a full, uneventful recovery. Histopathologic examination revealed mild splenomegaly with a ruptured capsule of undetermined cause.

**Conclusion:**

Spontaneous splenic rupture is a rare event that may lead to life-threatening hemorrhage if not diagnosed and treated quickly. Although the cause of this patient's case was unknown, atraumatic splenic rupture has been associated with a variety of infectious diseases and demonstrates some risks the active duty military population may face while on deployment. Having an awareness of these pathogens and their role in splenic rupture, clinicians caring for military personnel must be prepared to recognize and treat this potentially fatal complication.

## Introduction

Traumatic splenic rupture has been well documented, with an estimated 30% rate of occurrence after operatively managed blunt abdominal trauma [1]. Spontaneous rupture of the spleen, however, is a rare clinical entity, and in the absence of trauma, the diagnosis and treatment of this potentially fatal complication are often delayed. Spontaneous splenic rupture has been described in the setting of known pathology, most commonly infectious and neoplastic processes affecting the reticuloendothelial system [2]. Atraumatic rupture of the normal spleen has also been reported, although its legitimacy has long been debated [1-3]. Regardless of the mechanism, patients typically present with upper abdominal pain, classically referred to the left shoulder (Kehr's sign), with evidence of peritonitis and hemodynamic instability [3-5]. Here we present the case of a previously healthy 30-year-old Marine with atraumatic splenic rupture upon return from Iraq. Although no definitive cause was identified, the patient's clinical presentation suggested an infectious source. A variety of infectious pathogens are known to cause splenic enlargement and thus predispose to rupture. Several of these bacterial, viral and parasitic agents are endemic to the Middle East and nearby areas of military operations, placing deployed personnel in these regions at risk for exposure.

## Case presentation

A previously healthy 30-year-old Caucasian male active duty Marine reported abdominal pain, fevers and malaise on his return flight home from Iraq. The patient noted feeling well before redeployment and denied any injury or illness over the course of his six-month tour of duty, when he was stationed at a US Army installation near Baghdad. He denied any recent or remote history of trauma, including compression or blast injuries. He remained inside the base perimeter for the duration of deployment, had no contact with the local human or animal population and denied consuming local food or beverages. He reported using DEET (*N*,*N*-Diethyl-*meta*-toluamide) insect repellant and wearing permethrin-treated uniforms and denied insect bites. He was not aware of any sick contacts. Over the next 24 hours, the patient noted worsening abdominal pain and subjective fevers, as well as several episodes of watery diarrhea. He presented to the emergency department (ED) the morning after his arrival in the United States of America.

In the ED, the patient described his abdominal pain as diffuse and aching, unrelated to positional changes or oral intake. He denied anorexia, nausea and vomiting. His vital signs were stable and within normal limits, and the patient was afebrile at that time. Examination demonstrated mild tenderness to palpation in all quadrants without rebound or guarding. Bowel sounds were normal, his abdomen was soft and no organomegaly or distension was appreciated. The remainder of his physical examination was normal. The complete blood count (CBC) was unremarkable, with a white blood cell (WBC) count of 5,440/μL. The chemistry panel and urine analysis were normal. Liver function tests (LFTs) revealed mild transaminitis, with aspartate aminotransferase (AST) of 82 u/L and alanine aminotransferase (ALT) of 89 u/L but were otherwise unremarkable. The heterophile antibody test result was negative. Chest radiography results were normal. The appendix was not completely visualized on abdominal computed tomography (CT), but the study was otherwise unremarkable, with the remainder of bowel, liver, spleen and kidney appearance normal for technique. The patient was diagnosed with gastroenteritis at that time and discharged with conservative treatment.

Five days later, the patient returned to the ED. He reported resolution of diarrhea two days earlier but complained of persistent fevers and chills and worsening abdominal pain that was exacerbated by movement. He also reported a sore throat, rhinorrhea, cough and headache over the preceding two days, as well as occasional dizziness and lightheadedness. He noted one episode of nonbloody, nonbilious emesis three days earlier but otherwise denied nausea and vomiting. He reported mild anorexia but was tolerating a bland diet. He reported headache relief with ibuprofen and denied photophobia and neck stiffness. Vital signs were stable from his prior visit, with a temperature of 99.4°F. Mild bilateral anterior cervical lymphadenopathy was present, but the results of the head, eye, ear, nose and throat examination were otherwise unremarkable. Abdominal examination revealed increased tenderness to palpation in all quadrants but was otherwise unchanged from prior findings. He had normal rectal tone without evidence of bleeding, and guaiac testing results were negative. The remainder of his physical examination findings were normal. The CBC revealed a five-day decrease in hemoglobin and hematocrit, from 15.0 g/dL and 43.4% to 11.0 g/dL and 31.2%, respectively, and an increase in platelets from 105,000/μL to 200,000/μL. The WBC count was unchanged from the prior value. LFTs revealed an increase in AST to 111 u/L and ALT to 178 u/L. Review of urine cultures obtained in the ED five days prior revealed no growth. Repeat heterophile antibody testing results were negative. Flat and upright abdominal films revealed a nonspecific abnormal bowel gas pattern but were otherwise unremarkable. After evaluation and recommendations from general surgeons, the patient was admitted to the internal medicine service for further work-up and observation with serial abdominal examinations.

The following morning, the patient appeared pale, described worsening abdominal pain and orthostatic symptoms and was in obvious discomfort with movement. He was moderately tachycardic and tachypneic, with a temperature of 101.9°F. Abdominal examination revealed moderate distension, significantly increased and more localized tenderness to palpation in the left upper quadrant (LUQ), rebound tenderness and involuntary guarding. Serum laboratory testing demonstrated persistent transaminitis, increasing thrombocytosis and a further decrease in hemoglobin and hematocrit to 9.9 g/dL and 28.0%, respectively. Repeat abdominal CT demonstrated a fluid-filled abdomen consistent with hemoperitoneum, and after consultation with the surgeons, the patient was emergently taken to the operating room. Open exploratory laparotomy was performed via midline incision, with more than one litre of dark blood and clots visualized in the peritoneum, localized predominately in the LUQ. Inspection of the spleen revealed a tense, ruptured capsule with active bleeding. The gross appearance was otherwise normal, with no perisplenic adhesions visualized. The stomach, liver, small bowel and colon were examined, with no evidence of pathology. Splenectomy was performed without complication, with two units of packed red blood cells (RBCs) transfused intraoperatively. The patient recovered well postoperatively, with gradual resolution of symptoms and normalization of laboratory parameters. He was discharged on postoperative day seven with hemoglobin and hematocrit of 10.9 g/dL and 32.5%, respectively. He was started on daily aspirin for secondary thrombocytosis and received *Pneumococcus*, *Meningococcus *and *H. influenzae *immunizations before discharge.

Pathologic investigation was performed locally and by the Armed Forces Institute of Pathology Department of Hematopathology in consultation with the Department of Infectious Disease. Mild splenomegaly was noted, with the specimen weighing 321 g and measuring 14.1 × 9.2 × 6.1 cm. Grossly, the spleen was described to have a smooth, ruptured capsule of undetermined cause. The red pulp contained a diffuse, mixed infiltrate consisting of variable numbers of small lymphocytes, granulocytes, plasma cells and histiocytes. No infectious organisms were visualized with special staining (Grocott-Gomori methenamine silver nitrate, Brown and Brenn, Brown and Hopps, Fite, Warthin-Starry, periodic acid-Schiff with diastase, Ziehl-Nielsen), and immunohistochemical study results were normal. No significant atypia was identified. Results for serologic testing over the patient's hospital course and after discharge were negative throughout, including three blood cultures, two urine and throat cultures, stool culture, ova and parasites, fecal leukocytes, two malaria thick and thin smears, HIV-1 Antibody, hepatitis virus panel, two heterophile Antibody tests, Epstein-Barr Virus Antibody panel and two Leishmania spp. Antibody panels. Before discharge, the patient was offered bone marrow biopsy to further elucidate the cause of his splenic rupture; however, he declined the procedure. The patient currently remains on active duty and at 18-month follow-up reported doing well since discharge, with no known sequelae.

## Discussion

In 1861, Rokitansky first reported atraumatic splenic rupture in a leukemic patient, and in 1874, Atkinson first described the rupture of an apparently normal spleen [1,6]. Since then, several authors have attempted to further delineate the subtypes of atraumatic splenic rupture. It has been proposed that "true spontaneous" or "idiopathic" rupture refer only to spontaneous rupture of a normal spleen, and spontaneous rupture of a diseased spleen is most appropriately termed "pathologic" or "occult" rupture [3]. "True spontaneous" splenic rupture has been described in the literature, but its validity has often been challenged. Wright and Prigot stated, "There is no such clinical entity as spontaneous rupture of the normal spleen," [4], implying that thorough questioning and investigation will reveal a history of trauma or splenic pathology. After reviewing reports of spontaneous splenic rupture through 1958, Orloff and Peskin [2] found that most cases did have an identifiable pathologic or traumatic source. From this review, they proposed four criteria necessary to make the diagnosis, and in 1991, Crate and Payne [7] added a fifth condition (Appendix 1). Satisfying these criteria makes the diagnosis of "true spontaneous" splenic rupture challenging because investigators must carefully consider all potential causes. Several mechanisms have been proposed to explain atraumatic rupture in the absence of disease, primarily focusing on unrecognized sources of trauma or pathology (Appendix 2).

Atraumatic splenic rupture of the diseased spleen has been more commonly described and is associated with various infectious, neoplastic, hematologic, metabolic, inflammatory and local splenic disorders (Table 1). Although the cause of our patient's splenic rupture was undetermined, his clinical presentation was suggestive of an infectious process. As depicted in Table 1, a variety of bacterial, viral and parasitic agents have been reported to cause splenic enlargement and predispose to spontaneous rupture. Of these known pathogens, malaria in particular has been extensively studied because it is estimated to be the primary cause of spontaneous splenic rupture worldwide [8-11]. Its review illustrates some theories behind pathologic rupture of the spleen, as well as the unique infectious risks that our military personnel face while on deployment.

**Table 1:** Some Causes of Pathologic Splenic Rupture [1,3]

Infectious	Neoplastic and Hematologic
Bacterial	Leukemia
Staphylococci	Lymphoma
Streptococci	Myelofibrosis
*Clostridium *spp.	Multiple myeloma
Actinomycosis	Splenic malignancy
*Salmonella *spp.	Hepatocellular carcinoma
*Enterobacter *spp.	Hemophilia
*Campylobacter *spp.	Congenital factor XIII deficiency
*Haemophilus *spp.	Protein S deficiency
Tularemia	Hemolytic anemia
Brucellosis	Polycythemia
Legionellosis	Anticoagulant therapy
Tuberculosis	
Viral	Local splenic causes
Infectious mononucleosis	Splenic cyst
Mumps	Splenic vein thrombosis
Hepatitis A	Splenic peliosis
Dengue	Diffuse splenic angiomatosis
Cytomegalovirus	Portal hypertension
Rubella	Splenic infarct
Varicella-zoster	
Influenza	Miscellaneous
HIV	Sarcoidosis
Other	Amyloidosis
Malaria	Wilson's disease
Leishmaniasis (visceral)	Gaucher's disease
Syphilis	Cirrhosis
*Echinoccocus *spp.	Crohn's disease
Typhus	Polyarteritis nodosa
Leptospirosis	Systemic lupus erythematosus
Q fever	Pancreatitis
Relapsing fever	Rheumatoid arthritis
Candidiasis	Wegener's vasculitis

The World Health Organization estimates that 300 to 500 million humans worldwide have malaria each year, with an estimated 2% occurrence rate of splenic rupture and up to 80% mortality of these cases [3,8,9]. Rupture is most commonly seen with *Plasmodium vivax *infection; however, all four *Plasmodium *species have been implicated. Some theories propose that splenic congestion results from infiltration of red pulp by parasitized RBCs, pigment-laden macrophages and other WBCs; others cite the pooling of platelets as a causal factor [8]. However, with splenomegaly occurring in up to 90% of patients, typically within four days of symptom onset, most theories agree that rupture results from acute, rapid splenic congestion [9,10]. Regardless of species, the majority of ruptures occur with primary infection in nonimmune individuals, with lower rates among inhabitants of malarial endemic areas. This is thought to result from the gradual splenic enlargement and fibrosis seen in these populations, which likely protects from future rupture. In contrast, nonimmune patients, travelers in particular, have been shown to have significantly higher rates of splenic rupture with infection [8,10]. This point is of particular importance for our military personnel, who are often deployed in regions endemic for malaria and other pathogenic causes of splenic rupture and rarely with the protection of immunity.

Despite having a clinical presentation consistent with an infectious cause, our patient's microbiologic and histopathologic investigations did not reveal an infectious source, including *Plasmodium *spp. Based on the information at hand, he did meet the proposed criteria for spontaneous splenic rupture (Appendix 1). In retrospect, however, there were some variations in sampling methods and additional tests that may have helped identify a particular agent. If stool samples had been obtained at the time of the patient's diarrhea, it is likely that cultures would have been more revealing because the chances of growing out *Salmonella*, *Campylobacter*, *Shigella *and *Yersinia *spp. decrease rather quickly as diarrhea subsides, with culture yield markedly reduced after three days [12]. If bone marrow biopsy had been performed, in addition to excluding lymphoproliferative and neoplastic processes, it may have provided a medium for difficult-to-culture organisms (i.e., *Brucella*, *Leishmania*, *Mycobacteria *and *Histoplasma *spp. as well as *Salmonella typhi*, for which bone marrow cultures have been specifically recommended [13]). Additionally, culture of fresh splenic tissue may have proven useful in identifying a particular organism as a more sensitive alternative to special staining. However, in this case, the resected sample was delivered from the operating room in formalin, eliminating that possibility.

Although no infectious source was identified in this Marine and the cause of his splenic rupture was ultimately undetermined, the case does serve as a reminder of the infectious causes that pose a threat to deployed military personnel. Particularly as the U.S. and joint military focus shifts from operations in Iraq to Afghanistan and the United States Central Command's area of responsibility continues to broaden, it is increasingly vital to consider the infectious risks facing our troops. Of the infectious diseases known to cause pathologic splenic rupture, many are endemic to the Middle East and South-Central Asia (Table 2). Military members receive vaccinations for several of these pathogens, and some are common causes of respiratory and diarrheal illness at home and abroad. However, others are not as routinely encountered in everyday practice and thus may not be given sufficient consideration to make a timely diagnosis. Of particular concern, based on prevalence in the region, are malaria, visceral leishmaniasis and tuberculosis [14-17]. Realizing that our military personnel have significant exposure to these infectious causes of splenic rupture, physicians caring for them abroad and at home must consider their transmission, as well as this potentially fatal clinical entity.

**Table 2:** Some Infectious Causes of Pathologic Splenic Rupture that are Endemic to and of Particular Concern in the Middle East and South-Central Asia [14-17]

Bacterial	Viral	Other
Staphylococci	Mumps	Malaria
*Clostridium *spp.	Hepatitis A	Leishmaniasis (visceral)
*Salmonella *spp.	Dengue	Typhus
*Campylobacter *spp.	Rubella	Leptospirosis
*Haemophilus *spp.	Influenza	Q fever
Brucellosis	HIV	Relapsing fever
Tuberculosis		*Echinococcus *spp.

## Conclusion

Spontaneous splenic rupture is a rare event that, if unrecognized, may lead to life-threatening hemorrhage. Although the cause of our patient's rupture was not determined, the case serves to remind of the infectious causes of this condition that are faced by our deployed troops. Physicians caring for military personnel must be alert to these infectious risks and their association with splenic rupture because a high index of suspicion is required to promptly diagnose and treat this serious complication.

## Abbreviations

ALT: alanine aminotransferase; AST: aspartate aminotransferase; CBC: complete blood count; CT: computed tomography; DEET: *N*,*N*-Diethyl-*meta*-toluamide; EBV: Epstein-Barr virus; LFT: liver function test; LUQ: left upper quadrant; RBC: red blood cell; WBC: white blood cell.

## Consent

Written informed consent was obtained from the patient for publication of this case report. A copy of the written consent is available for review by the Editor-in-Chief of this journal.

## Competing interests

The authors declare that they have no competing interests.

## Authors' contributions

JPR and CMS both directly took part in patient care and contributed to data acquisition, research, manuscript conception, drafting and writing of the manuscript. JR was responsible for final editing and submission. Both authors have read and approved the final manuscript.

## Appendix 1 Criteria for Spontaneous Splenic Rupture

- No history of trauma or unusual physical effort, either before or on retrospect questioning after surgery.

- No evidence of disease that may affect the spleen.

- No evidence of perisplenic adhesions or scarring of the spleen to suggest trauma or previous trauma or rupture.

- Other than findings of rupture and hemorrhage, the spleen should be normal on gross inspection and histologic examination.

- Acute phase and convalescent sera should show no increase in viral antibody titers suggestive of recent infection with types associated in splenic involvement.

## Appendix 2 Proposed Mechanisms to Explain Idiopathic Rupture

- Localized involvement of the spleen with a pathologic process, which is no longer apparent on rupture

- Reflex spasm of splenic vein causing acute splenic congestion

- Portal venous congestion with chronic splenic congestion

- Abnormally mobile spleen producing torsion and finally rupture

- Rupture of a degenerative or aneurysmal splenic artery

- Forgotten or unnoticed trauma

- Sudden increase in abdominal pressure leading to rupture
